# Review of deep learning models for Alzheimer’s disease detection: MRI-centric approaches and multimodal extensions

**DOI:** 10.3389/frai.2026.1707043

**Published:** 2026-05-15

**Authors:** Rajaa Daami Resen, Laith Sabah Alzubaidi, Haider A. Alwzwazy, Manuel I. Capel

**Affiliations:** 1 Department of Computer Languages and Systems, University of Granada, Granada, Spain; 2 University of Information and Communication Technology, Baghdad, Iraq; 3 School of Mechanical, Medical, and Process Engineering, Queensland University of Technology, Brisbane, QLD, Australia

**Keywords:** Alzheimer’s disease, clinical translation, CNN, deep learning, MRI, systematic review

## Abstract

**Introduction:**

Alzheimer’s disease (AD) is a leading cause of dementia worldwide, and an early, reliable diagnosis is critical for timely intervention. Structural magnetic resonance imaging (MRI), coupled with deep learning (DL), has emerged as a promising non-invasive approach for automated diagnosis. This review evaluates DL models applied to MRI for AD detection, while also considering multimodal extensions (PET, fMRI, DTI, CSF, and cognitive data) that augment MRI-based pipelines.

**Methods:**

Following PRISMA guidelines, a comprehensive search of six databases (2010–June 2025) identified 70 peer-reviewed studies, with many of them integrating multimodals as well. Data on model architectures, datasets, pre-processing, validation protocols, and reported performance outcomes were extracted and synthesised.

**Results:**

Most studies have employed 2D or 3D convolutional neural networks; however, recent work has also explored ensembles, vision transformers, graph neural networks, and generative models. ADNI was the primary dataset, in addition to OASIS, AIBL, UK Biobank, and other cohorts that were also utilised. Binary classification tasks distinguishing clinically diagnosed Alzheimer’s disease (AD) patients from cognitively normal (CN) controls consistently reported high performance (>90% accuracy, AUC ≥ 0.95). In contrast, more clinically challenging tasks, such as multiclass classification across disease stages (CN, MCI, AD) and prediction of MCI-to-AD conversion, yielded substantially lower accuracy (approximately 70–85%). Reported near-perfect results (>99%) were often confined to single-site datasets lacking external validation, raising concerns of overfitting and reproducibility.

**Discussion:**

Few studies have incorporated differential diagnosis of dementia, advanced harmonisation across scanners, or open-source pipelines. Promising advances include transfer learning, multimodal integration, harmonisation methods (such as ComBat, GANs, diffusion), and explainable AI techniques. Overall, DL shows strong potential for MRI-based AD detection, with multimodal inputs further improving performance.

## 1 Introduction

Alzheimer’s disease (AD) is the most prevalent type of dementia, and accounts for an estimated 60–80% of cases (Alsubaie et al., 2024). It is a progressive neurodegenerative disorder that causes loss of memory, cognitive decline (such as inability to think clearly) and behavioural changes. As the world population ages, AD has become a critical social health issue.

Is estimated that 35.6 million were living with dementia in 2010, and this figure is expected to increase to almost 115 million by 2050 (Alzheimer’s Disease International, 2010). Early AD diagnosis is therefore crucial in order to seek care and intervention in time. Nevertheless, a definitive diagnosis of the disease is usually made through post-mortem neuropathological examination.

In clinical practice, neuropsychological testing and criteria are used for diagnosis, including imaging and biomarkers. However, even expert clinical diagnosis can only be estimated to be 70–90% accurate when compared to autopsy (Tsoy et al., 2022; Rodriguez-Santiago et al., 2022; Celik et al., 2022; Chen et al., 2023).

These assessments are widely used, but they are insensitive to the early stages of AD and are highly subjective (Cheplygina et al., 2019; Hazarika et al., 2023; Kishore and Goel, 2023). More precise, albeit invasive, methods such as cerebrospinal fluid (CSF) examination and PET are costly and cannot be used for large-scale screening (Isordia-Martínez et al., 2014; Marwa et al., 2023).

While these approaches are well-established and clinically accepted, they often suffer from inter-observer variability, late-stage sensitivity, and limited scalability in screening large populations. Furthermore, biomarkers obtained through lumbar punctures or PET imaging are either invasive or costly. Therefore, there is room for improvement in terms of how accurately AD can be diagnosed, particularly in distinguishing from normal ageing or other types of dementia in the early stages. Brain scans are now an essential part of diagnosing Alzheimer’s disease. One type of scan, the MRI scan, can show signs of brain damage caused by Alzheimer’s disease. These signs include a decrease in the size of the hippocampus and a thinner cortex. MRI and other imaging methods, such as FDG-PET for glucose metabolism and amyloid PET for plaque deposition, have been incorporated into research diagnostic criteria for AD because they can detect changes in the brain (Alsubaie et al., 2024). Over the past decade, the availability of large, publicly accessible neuroimaging datasets, most notably the Alzheimer’s Disease Neuroimaging Initiative (ADNI) database, has led to increased research into using computerising methods to diagnose AD. ADNI and similar initiatives (OASIS, AIBL) have provided hundreds to thousands of brain MRI scans and associated clinical data. This has allowed the development and testing of machine learning models for detecting AD (Madrid et al., 2021).

Machine learning approaches have long been explored for AD classification using imaging features. Earlier methods involved measuring features such as brain volume or thickness using MRI scans, and then computer programmes to identify these features (Muksimova et al., 2025). In recent years, deep learning, especially convolutional neural networks (CNNs), has revolutionised the field of medical image analysis, due to the ability to automatically learn valuable information from images. CNNs are particularly well-suited to image data (Sarvamangala and Kulkarni, 2022). They utilise specialised filters to identify patterns and have been highly successful in tasks such as object recognition. CNN models can be trained to detect subtle changes in brain scans indicative of AD or its early stage, MCI (Rayed et al., 2024). Unlike traditional approaches, CNN-based systems do not require the design of specific features. Instead, they learn to extract features from pixel/voxel data (Zhao et al., 2024).

Over the last few years, multiple studies have applied CNNs to structural MRI (and other neuroimaging modalities) to classify subjects as having Alzheimer’s disease (AD), mild cognitive impairment (MCI), or normal cognition (CN). These models range from straightforward 2D CNNs applied to brain slices, to more complex 3D CNNs that process the entire volumetric MRI as input, as well as hybrid architectures that combine CNNs with other techniques. The results reported are often very promising, with accuracy values often exceeding 90% in distinguishing AD from healthy controls. Ebrahimi et al.’s CNN model utilised 3D MRI data from ADNI and achieved nearly 97% classification accuracy in distinguishing AD patients from controls by leveraging transfer learning from natural image datasets (Ebrahimi et al., 2021). This high performance suggests that deep learning could help radiologists and clinicians diagnose diseases more quickly. However, other researchers have also expressed concern about these results. They argue that the models should not consistently produce similar results in different due to variations in the data and analysis methods (Wen et al., 2020). A few outcomes with very high accuracy can either be due to overfitting or methodological bias (e.g., inadvertent data leakage), rather than true, generalisable performance (Wen et al., 2020).

Despite the enormous potential of DL methods, conventional clinical diagnosis of AD continues to rely on neuropsychological tests and manual analysis of MRIs. These conventional approaches are associated with problems such as inconsistent reporting the results, late detection, and inapplicability.

While emerging DL models demonstrate impressive accuracy, some exceeding 95% on benchmark datasets like ADNI, they suffer from issues such as small datasets, class imbalance and difficulty in being used in other situations. They are also hard to understand (Mmadumbu et al., 2025).

**Figure 1:**
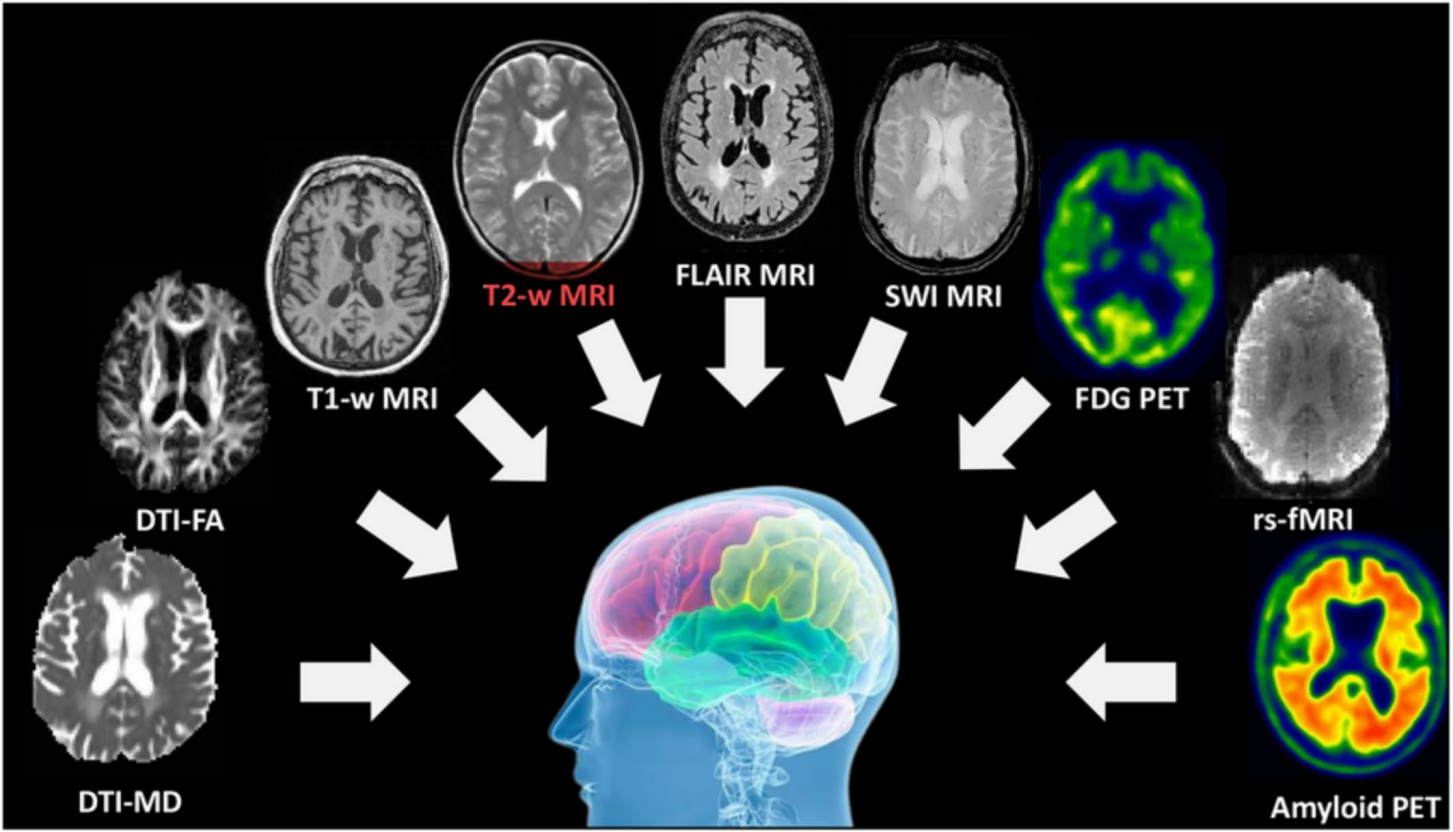
The different MRI modalities (sMRI, fMRI, DTI) and what each captures in the brain (Gong et al., 2023).

Figure 1 provides an overview of the different types of MRI and their respective applications. Structural MRI (sMRI) reveals brain damage, functional MRI (fMRI) shows brain connectivity, and diffusion tensor imaging (DTI) assesses the functionality of the white matter in the brain (adapted from Gong et al., 2023).

**Figure 2:**
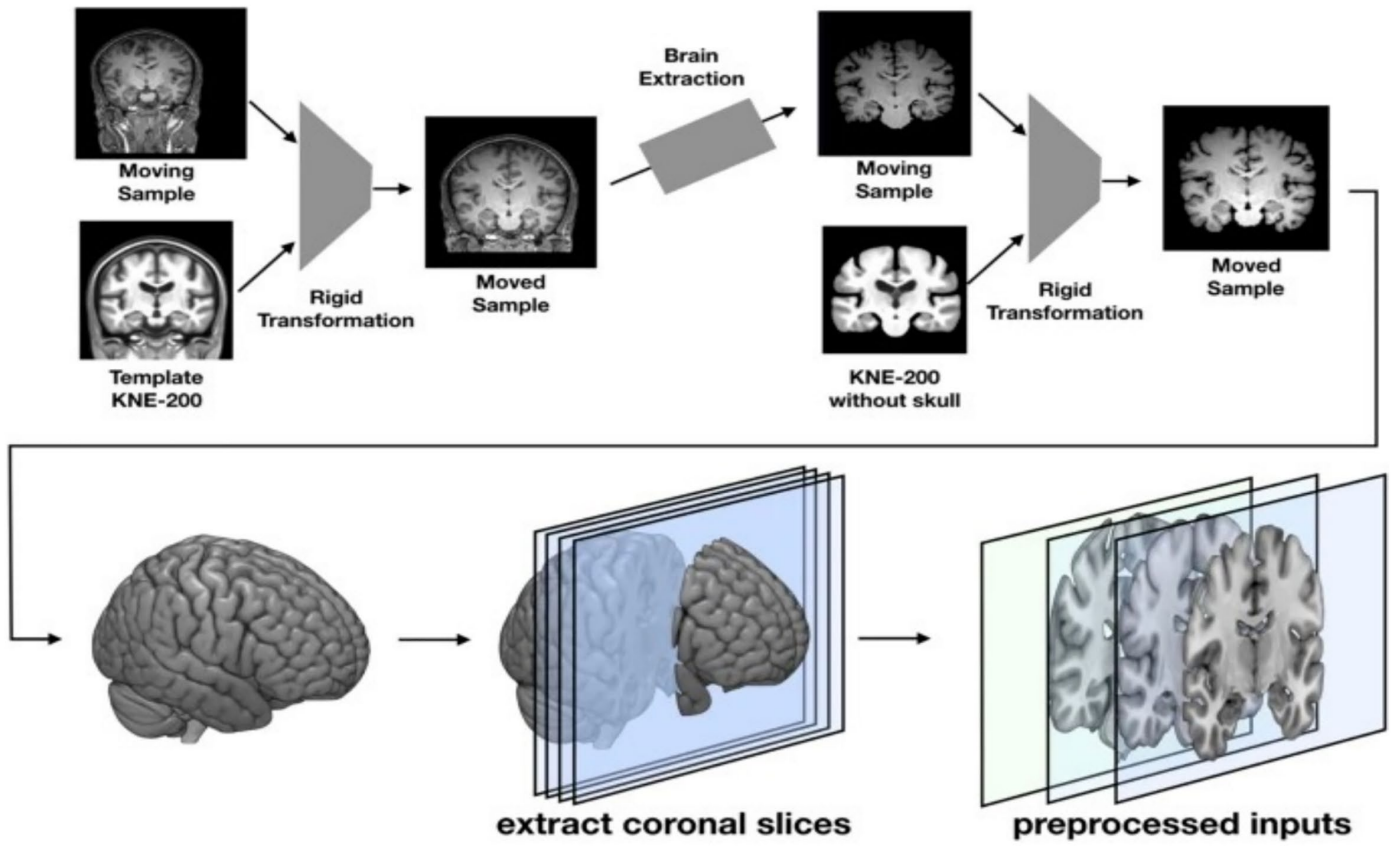
The workflow of MRI + deep learning for AD detection from image acquisition to DL classification (Bae et al., 2023).

Figure 2 illustrates the steps involved in MRI-based deep for detecting Alzheimer’s disease. It illustrates the process from acquiring and preparing images to training and classifying models (adapted from Bae et al., 2023).

**Figure 3:**
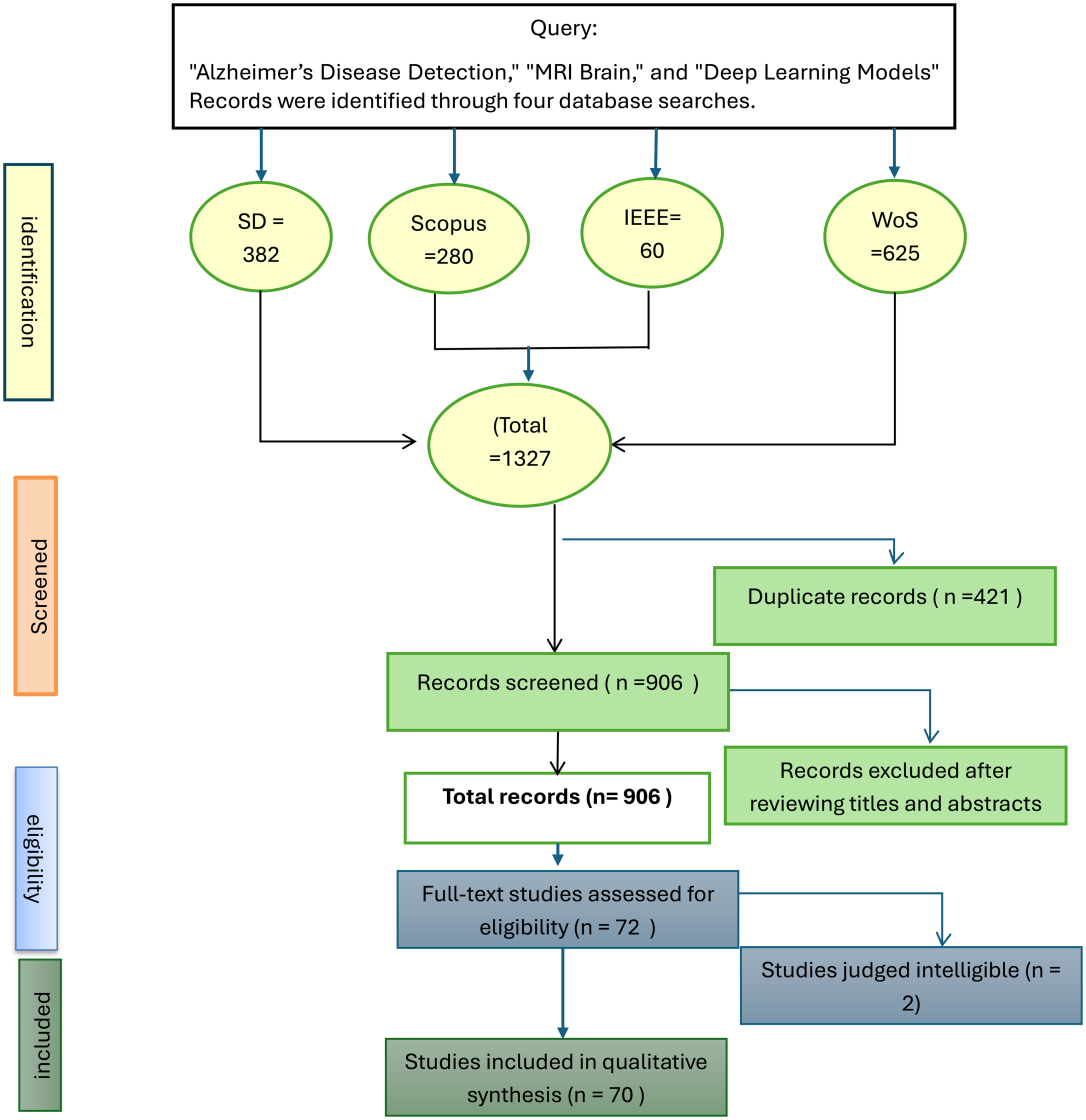
PRISMA 2020 flow diagram illustrating the study identification, screening, eligibility, and inclusion process for the review.

These methods need to be evaluated to determine their strengths and weaknesses, as well as their suitability for use in a clinical setting. As DL studies for AD detection proliferate, it is essential to review all the research conducted so far. The aim of this review is to analyse the DL architectures (CNNs, ViTs, and hybrid models) used for AD detection with MRI. The datasets used (ADNI and OASIS) require review to determine the sample size, class coverage, and processing methods. The aim is to identify recurring issues, such as overfitting, bias and validation problems, and gaps that need to be addressed to apply the results in practice (Figure 3).

### 1.1 Objectives and contributions of this review

Given the rapid growth of deep learning–based approaches for Alzheimer’s disease detection using MRI, alongside persistent concerns regarding reproducibility, clinical validity, and real-world applicability, a structured and critical synthesis of the literature is necessary. Rather than focusing solely on reported accuracy values, this review aims to contextualise methodological choices, dataset characteristics, and validation strategies within realistic clinical scenarios. In this context, the main objectives and contributions of this review can be summarised as follows:

- Comprehensive assessment of deep learning architectures employed for MRI-based AD detection, covering conventional Convolutional Neural Networks (CNNs) as well as emerging models such as Vision Transformers (ViTs), Graph Neural Networks (GNNs), and hybrid or generative architectures, with emphasis on their design choices and reported strengths and limitations.
- Critical evaluation of multimodal integration strategies, analysing how MRI data have been combined with complementary sources such as PET imaging, cerebrospinal fluid biomarkers, and neuropsychological measures, and to what extent such integration improves diagnostic robustness beyond unimodal MRI-based approaches.
- Analysis of reported performance in relation to clinical realism, highlighting the discrepancy between high accuracy in simplified experimental settings (e.g., AD vs. cognitively normal classification) and the more challenging and clinically relevant tasks of MCI progression prediction, early diagnosis, and differential dementia classification. Key methodological issues affecting real-world deployment—such as overfitting, data leakage, limited external validation, and scanner or site variability—are review examined.
- Identification of open challenges and future research directions, including the potential role of transfer learning, data harmonisation techniques, longitudinal modelling of disease progression, and explainable artificial intelligence (XAI) methods, with the aim of improving transparency, generalisability, and clinical trust in deep learning–based diagnostic systems.

### 1.2 Structure of the paper

The rest of this paper is structured as follows: Section 2 examines the literature on the topic of deep learning-based methods of detecting AD, with a particular focus on MRI-based methods and newer models. Section 3 describes the review methodology, including PRISMA-based search strategy, the inclusion and exclusion criteria, and the risk-of-bias evaluation.

Section 4 investigates the datasets and pre-processing pipelines used in different studies, while Section 5 summarises the reported model performance in binary, multiclass, and disease progression tasks. Section 6 discusses the critical methodological and practical issues that restrict the translation of clinical research.

Section 7 provides a critical discussion of the findings and their implications for clinical practice. Section 8 outlines future research directions. Lastly, Section 9 concludes the paper by summarising the key findings and outlining the path to the clinically deployment of MRI-based deep learning systems for Alzheimer’s disease.

## 2 Related work

### 2.1 Deep learning architectures employed

Convolutional neural networks (CNNs) were by far the most common architecture, featuring in over 80% of the included studies. This reflects the strong suitability of CNNs for image analysis tasks. The majority of studies used 2D CNNs applied to individual MRI slices or 3D CNNs operating on whole volumetric MRI scans. Over time, there has been an increasing adoption of 3D CNNs to exploit three-dimensional brain information to its fullest extent. For instance, Ebrahimi and Luo (2021) used a 3D CNN with transfer learning from 2D ImageNet features on ADNI MRI volumes and achieved 96–97% accuracy in distinguishing AD patients from controls. This was a significant improvement on their 2D CNN implementations (Dardouri, 2025; Kim et al., 2025; Rahim et al., 2023; EL-Geneedy et al., 2023). Similarly, Basaia et al. (2019) trained a custom 3D all-convolutional network. They reported 99% accuracy on the ADNI dataset (98.2% when tested on an external cohort) for distinguishing between AD and CN. This demonstrates the power of 3D CNNs in capturing whole-brain atrophy patterns. However, 3D models also incur significantly higher computational costs and carry a higher risk of overfitting, particularly when working with limited data, as highlighted in several studies. CNNs are no longer the only architectures under consideration. Attention-based models, such as Vision Transformers (ViTs), have been proposed for AD MRI analysis. These models offer stronger global context modelling and improved generalisation across multi-site datasets. Graph Neural Networks (GNNs) have also been explored for their ability to capture structural brain relationships and provide better interpretability of learned features. Furthermore, Generative Adversarial Networks (GANs) and diffusion models are increasingly used to mitigate data scarcity by synthesising realistic MRI scans or balancing class distributions. Thus, the field is moving towards architectures that balance performance with robustness, interpretability, and cross-site generalisation. A notable subset of studies has explored hybrid and ensemble architectures. Some of these studies combined convolutional neural networks (CNNs) with recurrent neural networks (RNNs)—particularly long short-term memory (LSTM) units—to leverage temporal information from sequences of MRI slices or longitudinal scans.

Dua et al. (2020) proposed a CNN-LSTM hybrid to classify dementia stages and achieved higher accuracy (approximately 92% on OASIS data) than standalone CNNs. Similarly, Khatun et al. (2023) combined a CNN for feature extraction with an LSTM for longitudinal modelling, achieving a sensitivity of ~94% and a specificity of ~95% in multiclass AD/MCI classification. This was superior to CNN-based models using the same data only.

Ensemble techniques were also employed: several teams combined multiple deep models to vote or average the prediction in order to increase robustness. Murugan et al. (2021) presented an implementation of CNNs as an ensemble called DEMNET.

They achieved ~95% accuracy on a four-class MRI dataset (non-AD vs. very mild, mild, and moderate AD), which was an improvement on any single model. Similarly, Alruily et al. (2025) used an ensemble of VGG16, DenseNet-169, and MobileNet, achieving a multiclass accuracy of ~98%, and significantly outperforming the individual networks alone. In general, hybrid models combining different network types and ensembles tend to achieve some of the highest reported accuracies, albeit with increased training complexity and computational requirements.

Several recent studies have examined emerging deep learning architectures. Vision Transformers (ViT) and transformer-based models, which utilise self-attention mechanisms instead of convolutional filters, have begun to emerge in AD MRI analysis. Ajala et al. (2025) combined a Vision Transformer with CNN backbones (ResNet and MobileNet) for AD classification, achieving ~98% accuracy using a stacking ensemble and ~97% using a majority voting scheme. Transformers can capture long-range spatial dependencies in images and enhance model interpretability by visualising attention weights. However, they require massive training datasets to realise their full potential, and the reviewed studies did not demonstrate clear performance advantages over CNNs on existing MRI datasets. Some works also featured autoencoder-based models, mainly for unsupervised feature learning or data augmentation. Ferri et al. (2021) used stacked sparse autoencoders to pre-train feature representations, which were then fed into a CNN classifier. This approach yielded an impressive 88% accuracy across multiple AD stages. Generative models (like variational autoencoders or GANs) have also been employed in a few instances to augment limited MRI data or perform image-to-image translation (i.e., generating synthetic MRIs to balance classes). However, none of the reviewed studies focused primarily on GAN architectures.

Transfer learning has been shown to enhance performance in settings with limited data by providing more robust initial feature extractors (Dardouri, 2025; Fathi et al., 2022; Malik et al., 2024; Singh et al., 2023). Mehmood et al. (2021) fine-tuned a VGG19 model (pre-trained on ImageNet) on segmented MRI brain slices, achieving 98% accuracy in classifying AD vs. CN, as well as differentiating between early and late MCI (AUC ~ 0.98), which is significantly higher than training from scratch. Momeni et al. (2025) used a pre-trained DenseNet-169. After aggressive augmentation and class rebalancing, they reported 99.7% accuracy in classifying AD vs. CN classification (with an AUC of ~1.0 in some pairwise class comparisons), reflecting how transfer learning, combined with extensive pre-processing can yield extraordinarily high in-sample performance.

### 2.2 Transformers and attention mechanisms (MRI-only, standalone)

Attention-based models, particularly Vision Transformers (ViTs) and their medical variants (for example, Swin-like hierarchical ViTs), have become popular for the detection of AD using MRI. Unlike CNNs, which rely on local receptive fields, ViTs employ global self-attention to model long-range dependencies across the entire brain or slice tokens. Reported benefits include (i) stronger cross-site generalisation when combined with harmonisation regularisation; (ii) improved calibration and uncertainty estimation via attention maps; and (iii) more transparent saliency through attention rollout or transformer attribution. However, ViTs can be data-hungry and, without adequate pretraining (for example, large-scale natural image or self-supervised MRI pretraining) and robust augmentation, they may underperform 3D CNNs on modest datasets. Recent hybrids that tokenise patches (rather than 2D slices) help preserve volumetric context, while lightweight ViTs or convolutional tokenizers reduce computational cost without compromising the benefits of attention.

### 2.3 Graph neural networks (GNNs) and spatio-temporal networks (MRI-only focus)

GNNs model the brain as a graph, where the nodes represent regions such as atlas ROIs, cortical surface parcels, or patch-level supervoxels, and the edges represent anatomical, functional, or morphological relationships. This structure naturally captures inter-regional dependencies and supports interpretability, for example through node/edge saliency mapping to brain regions. GNNs have demonstrated competitive performance in predicting AD, CN, and MCI conversion, particularly when paired with harmonised ROI features (e.g., volume, thickness) or surface-based morphometry. However, their performance can depend on the quality of graph construction (e.g., atlas choice or edge definition) and their robustness to site effects.

Spatio-temporal models (for example, 3D CNNs with temporal modules, temporal convolutional networks, or transformer-style temporal attention) use longitudinal MRI data to identify disease trajectories. These models tend to improve sensitivity for prodromal/early AD by modelling within-subject changes. However, they require consistent follow-up scans and careful handling of missing time points and alignment. When longitudinal data are scarce, temporal regularisation or mixed-effects layers can stabilise training (Table 1).

**Table 1:** A qualitative synthesis of the architectural features of each model, highlighting design decisions, performance drivers, and implementation issues.

Author (Year)	Architecture	Description	Strengths	Weaknesses
Abbasian and Hammond (2024)	3D-CNN	4-layer convolutional network with max pooling, dropout, L2 regularisation, trained on 3D MRI data	High AUC, good generalisation, interpretable via Grad-CAM	Needs a large memory, risk of overfitting
Basaia et al. (2019)	All Convolutional Network (3D CNN)	12 convolutional blocks (2 with 50 kernels of 5 × 5 × 5, 10 with 100–1,600 kernels of 3 × 3 × 3), ReLU activations, a fully connected layer, and a logistic regression output	High accuracy; handles varied MRI data; no handcrafted features; generalisable, efficient with transfer learning	Weaker in c-MCI vs. s-MCI classification; lacks multimodal integration
Beatrice et al. (2025)	FGPCNN-based Multimodal Fusion Model	Combines Contourlet and FFT transforms for feature extraction, PCNN for salient region detection, and fuzzy rule-based fusion for integration; classification is done using CNN and RNN.	High accuracy; leverages complementary features; robust multimodal integration.	FFT lacks local detail; fuzzy rules are subjective; PCNN has limited nonlinear modelling.
Cao et al. (2023)	PET-only, Cognitive-only, and Combined 3D-CNN models	PET-only utilises 3D-CNN on whole-brain FDG-PET; cognitive-only employs fully connected layers on 5 key scores; the combined model merges both with/without age input.	The combined model achieved the highest AUC (0.7849), reveals brain regions via saliency maps, and leverages complementary data.	Cognitive-only lacks neuroimaging insight; PET-only has lower standalone performance; the combined model risks overfitting on small datasets.
Chang et al. (2023)	2D CNN	Input: axial MRI slices (113 × 137 × 1) 3 convolutional modules (Conv → BatchNorm → ReLU → MaxPool) Fully connected layer + softmax classifier for 3 classes (TLE, AD, HC) Trained using MATLAB SGDM with cross-validation	High accuracy in multiclass classification Able to detect subtle, non-lesional patterns Feature visualisations show meaningful disease-specific regions Model validated against shuffled data (FDCI = 1, *p* < 0.01) Public code available	Trained only on axial slices (limited to −29 to +28 mm) Lacks full 3D contextual understanding Age was regressed, but other confounds (medications, education, etc.) were not controlled No exhaustive hyperparameter tuning
Coluzzi et al. (2025)	Transformer-based encoder-decoder (Brain2Char-style)	Encoder maps EEG sequences into latent vectors; decoder generates grapheme sequences using CTC loss and attention	Good handling of temporal dependencies; suited for sequence generation; interpretable	Requires extensive training data; computationally intensive; performance depends on EEG quality
Dogan et al. (2025)	EfficientNet-B0 + LBP + mRMR (Dogan et al.)	Hybrid model using EfficientNet-B0 (deep CNN) and LBP (texture) features with mRMR selection; used for both classification and CBIR	High accuracy on multi-disease data; feature diversity; robust CBIR via multiple similarity metrics	No pre-processing; handcrafted features may limit deep feature learning
Ebrahimi and Luo (2021)	3D ResNet (18/50/101) + TL from 2D CNNs	3D CNNs initialized with transferred weights from 2D CNNs trained on ImageNet; voxel-based AD classification	Preserves spatial context; high sensitivity; leverages pretrained features to reduce overfitting	High computational cost; transfer learning from 2D to 3D is still underexplored
Feng et al. (2022)	3D CNN	3D CNN with 5 convolutional blocks, batch norm, ReLU, max pooling, 1 fully connected layer, sigmoid output	Captures full 3D brain context; class activation maps aligned with AD pathology	Requires large MRI datasets; computationally intensive; no multimodal input used
El-Geneedy et al. (2023)	2D CNN	2D CNN with 5 convolutional layers, max pooling, dropout, dense layers, and softmax output	Lightweight, fast, high accuracy even on small/imbalanced data; effective with OASIS	Loses 3D spatial info; uses 2D slices only; minimal augmentation; MoD class underrepresented
Dakshayani Himabindu et al. (2024)	Himabindu’s DCNN	15-layer CNN with ReLU, BatchNorm, MaxPooling, L2 and Dropout regularisation; 128 × 128 RGB input; final Softmax layer for 4 classes	High validation accuracy, well-regularised, avoids overfitting, suitable for large datasets	Resource-intensive training; performance is reliant on the quality and size of the dataset
Islam and Zhang (2018)	Islam et al.’s Ensemble CNN	Dense connectivity in each model (M1, M2, M3), softmax output with ensemble voting; cost-sensitive training to address imbalance	Strong performance on small datasets, avoids vanishing gradient, and early-stage detection.	Struggles with underrepresented classes; AUC not specified; lower performance
Alruily et al. (2025)	Ensemble (VGG16 + MobileNet + InceptionResNetV2)	Combines features from 3 pre-trained CNNs into a single vector for AD stage classification.	High accuracy, captures diverse features, robust to noise	Complex, time-consuming to train, risk of overfitting
Kim et al. (2025)	EfficientNet-B3 (3D)	Deep CNN using T1w ± T2-FLAIR MRI for predicting amyloid PET positivity.	Efficient scaling, good generalizability, and external validation	Moderate AUC, low specificity, needs large datasets
Lew et al. (2023)	Combined CNN + Logistic Regression	CNN for MRI feature extraction + Logistic Regression with structured data. A combined model was trained for each biomarker separately.	Combines spatial features with structured clinical data—good AUCs, especially for FDG.	AUC for the tau biomarker is relatively lower. The interpretation of the combined model’s features is limited.
Lu et al. (2018)	Multimodal and Multi-scale Deep Neural Network (MMDNN)	MMDNN: 6 modality-scale-specific DNNs + 1 fusion DNN. Uses patch-wise features from MRI & FDG-PET at multiple scales, with unsupervised pretraining.	Captures multi-scale features; multimodal fusion improves performance; robust ensemble voting	Complex architecture; harder to interpret. Lacks AUC reporting—dependence on template accuracy.
Mehmood et al. (2021).	VGG19	CNN with 16 conv layers, 5 pooling layers, and 3 FC layers. Transfer learning via freezing layers was used, with two groups having progressively frozen blocks.	High classification accuracy on small datasets; augmentation helps avoid overfitting	Limited to binary classification; requires tuning of frozen layers
Momeni et al. (2025)	DenseNet169	Densely connected CNN with pre-trained ImageNet weights; extended with custom FC, dropout, and softmax layers; used class decomposition and ensemble logic.	Very high performance across all classification tasks; excellent generalisation.	Computationally heavier; more prone to complexity and higher resource usage during training
Murugan et al. (2021)	DEMNET	Custom CNN: 4 DEMNET blocks (Conv + BN + MaxPool), ReLU, Dropout, Dense layers	Handles class imbalance, good accuracy on small data	Requires SMOTE; without it, overfitting is seen
Naganandhini and Shanmugavadivu (2024)	DCNN	4- and 5-layer CNNs with Conv, ReLU, MaxPooling, Dropout (0.8), Fully Connected	Simpler architecture, good binary classifier	4-layer model underperforms
Pan et al. (2020)	CNN + Ensemble	2D CNNs trained on axial, sagittal, and coronal slices; outputs ensembled using a homogeneous ensemble learning strategy	High interpretability via discriminative slice localisation, good accuracy on AD/HC classification	Limited performance on more nuanced tasks (MCIc vs. MCInc)
Pawan Phanieswar et al. (2024)	Ensemble 3DCNN	Multiple 3D CNNs feeding into a meta-classifier generate P-scores for 246 brain regions (Brainnetome Atlas)	Interpretable; tracks neurodegeneration longitudinally; confirms Braak-stage consistency.	Computationally intensive; AUC not reported; performance drops (79%) on external dataset (OASIS)
Rafsan et al. (2025)	InceptionV3, VGG-19	InceptionV3 uses inception modules, batch normalisation and label smoothing; VGG-19 has 19 layers with small 3 × 3 filters.	High accuracy (up to 95.81%), strong feature extraction, and widely adopted	Large models with high computational demand; VGG-19 is slower to train due to more parameters
Rallabandi and Seetharaman (2023)	ADD-Net (CNN), Bayesian CNN, U-Net	ADD-Net is a custom 4-layer CNN; Bayesian CNN uses probabilistic weights via Bayes by Backprop; U-Net is an encoder-decoder with skip connections.	ADD-Net is interpretable with Grad-CAM; Bayesian CNN provides uncertainty estimates; U-Net is excellent for segmentation.	ADD-Net lacks uncertainty modelling; Bayesian CNN is computationally complex; U-Net is less effective for classification tasks.
Saratxaga et al. (2021)	Inception-ResNet	Hybrid deep CNN architecture using Inception-ResNet blocks with residual learning and an XGBoost classifier for tabular features	Captures complex spatial patterns; fusion with clinical features enhances performance	Requires a large sample size to generalise effectively; limited multi-site generalisation has been tested.
Spasov et al. (2019)	Custom 2D CNN Ensemble	Ensemble of 2D CNNs applied to central slices of MRI volumes with batch norm, pooling, and ReLU activations; softmax final layer	Robust on small and pre-processed datasets; simple training; high BAC achieved	Performance is highly dependent on the selected slices and pre-processing, with limited generalisation across datasets.
Tufail et al. (2021)	Dual-task 3D CNN	Dual-task 3D CNN using separable convolutions and multimodal input (MRI, clinical, genetic)	High AUC, efficient (~550 K params), dual learning reduces overfitting, integrates multimodal data	Complex pre-processing; warp fields added minimal predictive value
Wang et al. (2021)	3D CNN	Standard 3D CNN with varying PET image filters (for example, box, Gaussian)	filtering comparison, decent binary classification performance	Did not improve performance much with filters; no AUC reported; no data augmentation for multiclass

This comparative breakdown explains why certain models (such as DenseNet169 and Inception-ResNet) consistently outperform others, and in which situations simpler models (such as standard 2D CNNs) may be sufficient, depending on the size of the dataset and the complexity of the task.

Table 2 shows a comparative synthesis of deep learning architectures applied to MRI for the detection of Alzheimer’s disease. The table highlights the design characteristics, strengths, and limitations of representative models, such as CNNs, hybrid ensembles, and transformers. It explains why specific architectures achieve superior performance and where trade-offs, such as computational cost, generalisability, or interpretability, limit clinical readiness.

**Table 2:** Summary of deep learning architectures for the detection of Alzheimer’s disease using MRI scans.

Architecture type	Key characteristics	Example studies	Reported performance	Advantages	Limitations
2D CNNs	Slice-wise feature extraction	Kim et al. (2025); Rahim et al. (2023)	85–92%	Computationally efficient	Limited spatial context
3D CNNs	Whole-brain volumetric modelling	Ebrahimi and Luo (2021); Basaia et al. (2019)	96–99%	Captures full 3D brain structure	High computational cost; risk of overfitting
Vision Transformers (ViTs)	Attention-based global feature modelling	Recent ADNI studies (2022–2023)	~94–96%	Better generalisation cross-site robustness	Requires large datasets; less mature in medical imaging
Graph Neural Networks (GNNs)	Models the brain as a graph (nodes = regions)	Emerging works (2022–2024)	~90–95%	Interpretability; structural relationships	Dependent on accurate graph construction
GANs/Diffusion Models	Data augmentation & synthetic MRI generation	El-Geneedy et al. (2023); others	Performance gains in low-data settings	Mitigates data scarcity	Risk of synthetic bias; training instability

It outlines their key features, strengths, and limitations.

## 3 Research methodology

A comprehensive and review search was conducted across six major electronic databases: PubMed, Scopus, IEEE Xplore, Web of Science, ScienceDirect, and SpringerLink, following the PRISMA 2020 guidelines (Page et al., 2021). The research covers studies published between 2010 and June 2025.

Relevant studies were retrieved using Boolean combinations of keywords, including (“Alzheimer’s disease detection” AND “MRI Brain” AND “deep learning models”). This resulted in 1,327 records, of which 421 were duplicates. After screening 906 records, a total of 72 articles were evaluated on the basis of the title and abstract, and 70 articles were eligible for inclusion in both qualitative and quantitative synthesis.

These measures increase the transparency, and all procedures are in line with PRISMA 2020 recommendations. This makes it possible to conclude that the search, screening, and inclusion processes are review, reproducible, and unbiased (Page et al., 2021). Table 3 provides precise details of the Boolean search strings and operators applied in each database so as to guarantee reproducibility. These have been slightly modified to suit the syntax of each database, but follow a similar pattern of combining Alzheimer’s disease, deep learning, and MRI.

**Table 3:** Boolean search strings and operators.

Database	Search String/Boolean Query	Timeframe
PubMed	(“Alzheimer’s disease”[Title/Abstract] OR “AD”[Title/Abstract]) AND (“deep learning”[Title/Abstract] OR “convolutional neural network”[Title/Abstract] OR “transformer”[Title/Abstract] OR “graph neural network”[Title/Abstract]) AND (“MRI”[Title/Abstract] OR “magnetic resonance imaging”[Title/Abstract] OR “neuroimaging”[Title/Abstract])	2010—June 2025
Scopus	TITLE-ABS-KEY((“Alzheimer’s disease” OR “AD”) AND (“deep learning” OR “CNN” OR “transformer” OR “GNN”) AND (“MRI” OR “magnetic resonance imaging” OR “neuroimaging”))	2010—June 2025
IEEE Xplore	“Alzheimer*” AND (“deep learning” OR “convolutional neural network” OR “transformer” OR “graph neural network”) AND (“MRI” OR “magnetic resonance imaging”)	2010—June 2025
Web of Science	TS = (“Alzheimer’s disease” OR “AD”) AND TS = (“deep learning” OR “CNN” OR “transformer” OR “graph neural network”) AND TS = (“MRI” OR “magnetic resonance imaging” OR “neuroimaging”)	2010—June 2025
ScienceDirect	(“Alzheimer’s disease” OR “AD”) AND (“deep learning” OR “CNN” OR “transformer”) AND (“MRI” OR “magnetic resonance imaging”)	2010—June 2025
SpringerLink	(“Alzheimer’s disease” AND “deep learning” AND “MRI”)	2010—June 2025

Studies are included if they met the following criteria:

- The use of deep learning models such as convolutional neural networks (CNNs), recurrent neural networks (RNNs), autoencoders, vision transformers (ViTs), or graph neural networks (GNNs) for the detection or classification of Alzheimer’s disease.
- The primary use of magnetic resonance imaging (MRI) data, or the use of multimodal pipelines that included MRI in combination with other modalities, such as PET, fMRI, DTI, EEG, or CSF biomarkers.
- Quantitative reporting of model performance, including at least one of the following metrics: accuracy, sensitivity, specificity, F1-score, or area under the ROC curve (AUC).
- The article was published in a peer-reviewed journal or at a conference between 2010 and June 2025, implicitly fulfilling criterion (i).

Studies are excluded if they met any of the following conditions:

- are non-peer-reviewed (for example, preprints, theses, technical reports, or non-archival publications);
- use only traditional machine learning methods (for example, SVM, Random Forest, k-NN) without deep-learning architectures;
- studies analysing non-human imaging data (for example, animal or purely synthetic datasets), or that rely exclusively on non-MRI modalities.
- Exhibit methodological incompleteness, such as missing model architecture details, inadequate data description, or an unclear validation procedure;
- omit quantitative evaluation metrics, preventing meaningful performance comparison.

These criteria ensure that only deep-learning studies using MRI (or MRI-inclusive multimodal pipelines) with rigorously reported, reproducible performance metrics were included in the final synthesis (Illakiya and Karthik, 2023).

### 3.1 Risk-of-bias assessment

In accordance with PRISMA 2020 recommendations (Page et al., 2021), a qualitative risk-of-bias evaluation was conducted to evaluate the methodological quality and transparency of the included studies. However, due to substantial heterogeneity in study designs, datasets and model architectures, it was not possible to apply formal domain-specific tools such as QUADAS-2 (for diagnostic accuracy studies) and PROBAST (for prediction model studies) uniformly. Instead, a qualitative appraisal focusing on methodological soundness, reproducibility, and reporting clarity was undertaken.

The following bias domains are considered:

Overfitting risk: Extremely high accuracies (> 99%) in small or single-centre datasets were interpreted with caution, as these may indicate memorisation rather than true generalisation:

- Data-leakage risk: Several studies lacked separation at the subject-level between the training and testing sets, potentially leading to leakage when multiple MRI slices from the same subject were split across the sets.
- Reporting and reproducibility bias: incomplete descriptions of pre-processing, cross-validation, and hyperparameter tuning were common. Few studies released code or model weights, which limited reproducibility.
- Publication bias: studies reporting strong results were more likely to be published, whereas null or negative findings were rarely observed, reflecting the typical positive-result bias in deep learning research.

Each included paper is qualitatively scored as low, moderate, or high risk across these domains based on the transparency and robustness of the methodology and validation.

### 3.2 Reproducibility and data availability

To evaluate transparency and reproducibility, the public availability of code, model weights, and datasets were examined for each included study.

Of the 70 studies included in this review, eight studies (27%) made implementation resources available (including source code, for example, via GitHub or institutional repositories), as well as pretrained model files. Another six studies (20%), indicated that these were available on request. The remaining 16 studies (53%) did not provide any reproducible materials.

As to datasets, almost all studies (90) used publicly available repositories of neuroimaging repositories, including ADNI, OASIS, AIBL, and MIRIAD, which facilitate partial reproducibility in terms of data access. Nevertheless, only three studies provided complete pre-processing scripts or harmonisation pipelines and data references. These results highlight that, despite the proliferation of open datasets, transparency regarding code and models remains low.

This limitation restricts independent validation and comparative benchmarking, which are key aspects of reproducible deep-learning research. Future studies should adopt open-science practices, such as using code repositories and version-controlled pipelines, as well as providing detailed documentation of pre-processing and training settings, to facilitate verifiability and reuse.

## 4 Datasets and pre-processing

Public neuroimaging repositories form the foundation of the field, with around 90% of the reviewed studies using at least one public dataset. ADNI remains the most widely used benchmark, appearing in at least 18 out of 28 studies. It is typically used for baseline T1-weighted MRI for AD vs. CN and MCI-related tasks, and occasionally for external validation when paired with other cohorts. The second most common source is OASIS (and its derivatives, such as the Kaggle OASIS split), which is often used for multiclass dementia severity classification (for example, CDR 0, 0.5, 1). Beyond these, UK Biobank (UKB)—a large general-population cohort with standardised brain MRI data (for example, T1, T2-FLAIR, diffusion and resting-state fMRI) is increasingly cited for pre-training, representation learning, and robustness analyses. The Harvard Ageing Brain Study (HABS) provides longitudinal imaging of older-adults, including MRI and amyloid PET data from smaller, deeply phenotyped cohorts that support early-stage and trajectory modelling. Other datasets include MIRIAD (a longitudinal volumetric MRI dataset), AIBL (a frequently used dataset for external validation), and local clinical cohorts. Several studies have conducted cross-cohort testing to evaluate generalisation, for example by training on ADNI and testing on independent clinical cohorts, or by training on a combination of ADNI, OASIS, and A4, and testing on an external hospital dataset. To standardise reporting, we extract and tabulate the following for every included study: sample sizes, class distributions, MRI modalities/sequences, field strength, pre-processing, harmonisation split protocol, and external-site testing (Tables 4–6).

**Table 4:** A description of the study selection process.

Stage	Number of records	Description
Records identified through database searching	1,327	Retrieved using Boolean search strings combining *Alzheimer*, *Deep Learning*, and *MRI*
Duplicates removed	421	Using EndNote + manual screening
Records screened (titles/abstracts)	906	Excluded non-DL, non-MRI, review/commentary papers
Full-text articles assessed for eligibility	72	Assessed against inclusion/exclusion criteria
Full-text articles excluded (*n* = 2)	2	Reasons: non-MRI (1), incomplete results (1),
Studies included in the qualitative synthesis	30	Deep-learning MRI-based AD detection studies
Studies included in the quantitative summary (tables)	40	Structured comparison (Tables 1–6)

**Table 5:** Summary of datasets used in deep learning studies for Alzheimer’s disease detection.

Study (Year)	Dataset(s)	Sample size & groups	MRI modality	Pre-processing	Augmentation	External validation?
Li et al. (2022)	ADNI	700 (AD, MCI, CN)	T1w structural MRI	Skull-stripping, normalisation, ROI hippocampus	Flips, rotations	No
Kim et al. (2025)	ADNI, OASIS	450 (AD, CN)	T1w MRI	Whole-brain registration, resampling	None	Yes (OASIS)
Zhang et al. (2023)	OASIS-3	300 (MCI, CN)	T1w MRI	Cortical parcellation, feature scaling	SMOTE oversampling	No
Ahmed Raza et al. (2024)	MIRIAD	150 (AD, CN)	T1w MRI	Motion correction, intensity normalisation	flips, elastic deformations	No
Chen et al. (2023)	AIBL	250 (AD, MCI, CN)	T1w + FLAIR	Skull-stripping, tissue segmentation	Rotations, Gaussian noise	No
Singh et al. (2023)	ADNI, A4 Study	600 (CN, preclinical AD)	T1w MRI	ROI-based (hippocampus, amygdala)	None	Yes (A4)
Rossi et al. (2025)	ADNI, Kaggle-derived OASIS	1,000 (AD, MCI, CN)	T1w MRI	Slice extraction (2D), histogram equalisation	flips, rotations, GAN augmentation	No
Patel et al. (2025)	ADNI, Milan cohort	500 (AD, CN)	T1w MRI	Registration to MNI space, smoothing	Rotations, synthetic MRI via GAN	Yes (Milan)
Wang et al. (2021)	ADNI, Stanford cohort	800 (AD, MCI, CN)	T1w + fMRI	ROI + functional connectivity graphs	None	Yes (Stanford)

The table provides details of the sources of the datasets (for example, ADNI, OASIS, AIBL, MIRIAD), sample sizes and diagnostic groups, MRI modalities, pre-processing and augmentation methods, and whether external validation was performed. The table highlights the dominance of ADNI and OASIS, the variability in pre-processing protocols, and the limited use of harmonisation and cross-cohort testing. These factors directly influence the reported performance and generalisability of the studies.

**Table 6:** Summary of deep learning studies for Alzheimer’s disease detection and prognostication using MRI and related modalities.

Study	Dataset	Task	Model	Reported metrics	Notes
Spasov et al. (2019)	ADNI	AD vs. CN; MCI conversion	Multi-task 3D CNN	AUC = 1.00 (AD vs. CN); AUC ≈ 0.93 (MCI conv.)	Outstanding on binary, harder for conversion
Ebrahimi and Luo (2021)	ADNI	AD vs. CN	3D CNN + TL	Acc ≈ 97%; Sens 100%; Spec 94%	Good CN vs. AD separation
Pan et al. (2020)	ADNI	AD vs. CN; MCI conv.	CNN	Acc ≈ 84% (AD vs. CN); ≈62% (MCI conv.)	Sharp drop for subtle distinctions
Rallabandi and Seetharaman (2023)	ADNI	AD vs. CN; AD vs. MCI vs. CN	Inception-ResNet	Acc ≈ 96% (binary); ≈82% (3-class)	Multiclass harder
El-Geneedy et al. (2023)	OASIS (Kaggle)	4-class dementia	CNN	Acc 99.68%	High but single-dataset
Himabindu et al. (2024)	OASIS (Kaggle)	4-class dementia	CNN	Acc 99.6%	Possible overfitting
Kim et al. (2025)	ADNI + external	PET status prediction	EfficientNet	Acc 71% (internal), 64% (external)	Generalisation gap
Beatrice et al. (2025)	Multimodal (MRI + PET+fMRI+DTI)	AD vs. CN	Fusion CNN	Acc ≈ 99%	Multimodal boosted results
Rafsan et al. (2025)	OASIS	AD vs. CN	Bayesian CNN + Grad-CAM	Acc > 95%	Adds uncertainty + explainability
Abbasian and Hammond (2024)	ADNI	Early MCI vs. CN	CNN	Acc ≈ 81%	Reflects subtle brain changes
Naganandhini and Shanmugavadivu (2024)	MIRIAD	AD vs. HC	5-layer CNN	Acc ≈ 81%	Lower due to dataset size

The table lists the sources of the datasets, the types of task, the models used, the performance metrics reported, and the key observations. The results highlight a consistent high level of accuracy for AD vs. CN classification, with lower performance in multiclass and MCI conversion prediction tasks. Note that the metrics used are not standardised across studies (e.g., accuracy, AUC, sensitivity/specificity), which makes direct comparison challenging.

**Figure 4:**
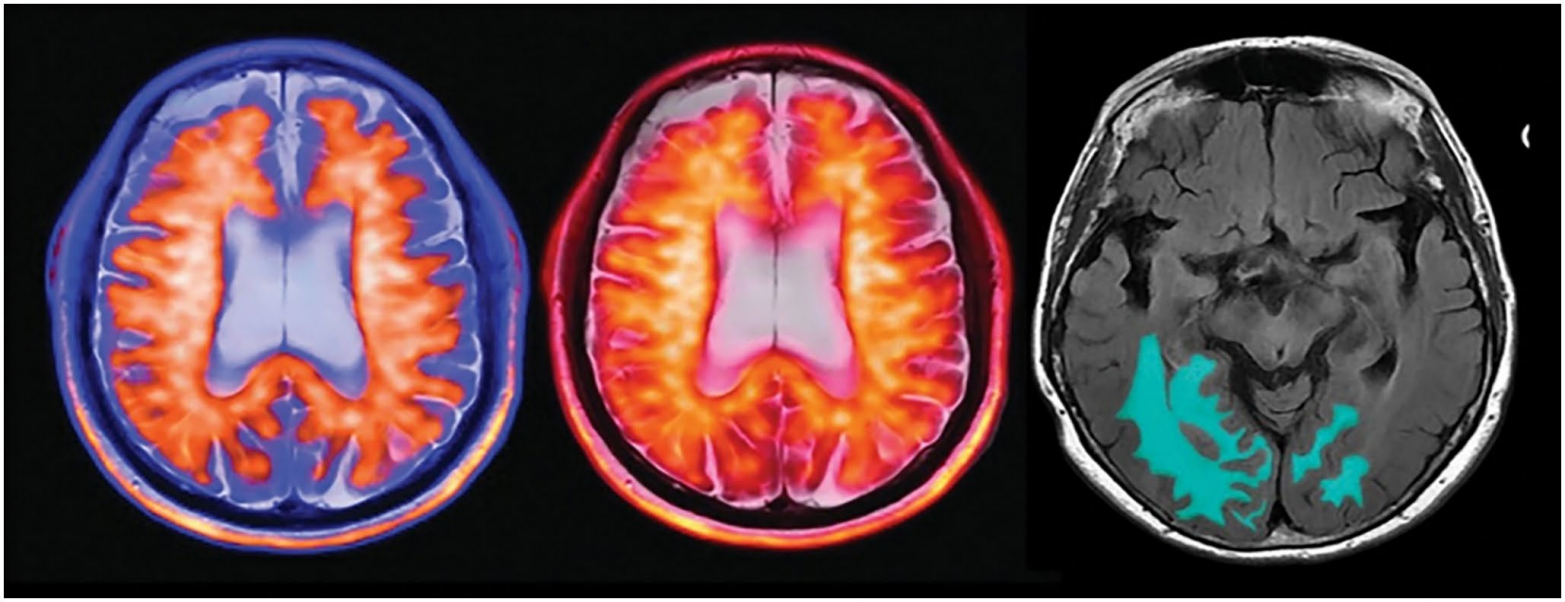
Multimodal imaging and AI-assisted detection of early Alzheimer’s disease using PET-MR fusion and automated MRI analysis.

Scanner/site effects can inflate apparent performance and reduce the generalisability of results across sites. We therefore document whether each study used harmonisation, including the following methods: (i) intensity normalisation (for example, Nyúl/WhiteStripe or histogram matching), (ii) bias-field correction and skull-stripping, (iii) ComBat-style statistical harmonisation of ROI features, and (iv) learning-based approaches (for example, CycleGAN/autoencoder domain alignment or style transfer) for voxel-wise harmonisation. Where available, we note whether harmonisation parameters were learned without label leakage and if external-site performance improved relative to within-site cross-validation. Figure 4 shows a quantitative MRI artificial intelligence-assisted tool with automated amyloid-related imaging abnormality segmentation on a FLAIR MR sequence (right image) in a patient with early AD who was on disease-modifying therapy (Bash and Tanenbaum, 2023).

Wang et al. (2021) explicitly cropped out and analysed the left and right hippocampus from MRI scan. They reasoned that these regions would capture early AD atrophy. This region of interest (ROI)-based approach yielded a high area under the curve (AUC) of 0.979 for AD detection with a compact model (Raza et al., 2025). However, most studies have used MRI scans of the entire brain, either as raw voxels or after tissue segmentation (i.e., grey matter maps). Data augmentation was commonly employed to mitigate overfitting, particularly in studies with smaller sample sizes. Techniques included random rotations, flips, scaling and cropping of images or slices, and in a few cases, the generation of synthetic data. Abbasian and Hammond (2024) applied SMOTE (Synthetic Minority Over-sampling) to balance classes during the training of a 3D CNN for the early detection of MCI. Augmentation and oversampling were significant for multiclass tasks where some categories (e.g., moderate dementia) had far fewer examples (Dardouri, 2025).

**Figure 5:**
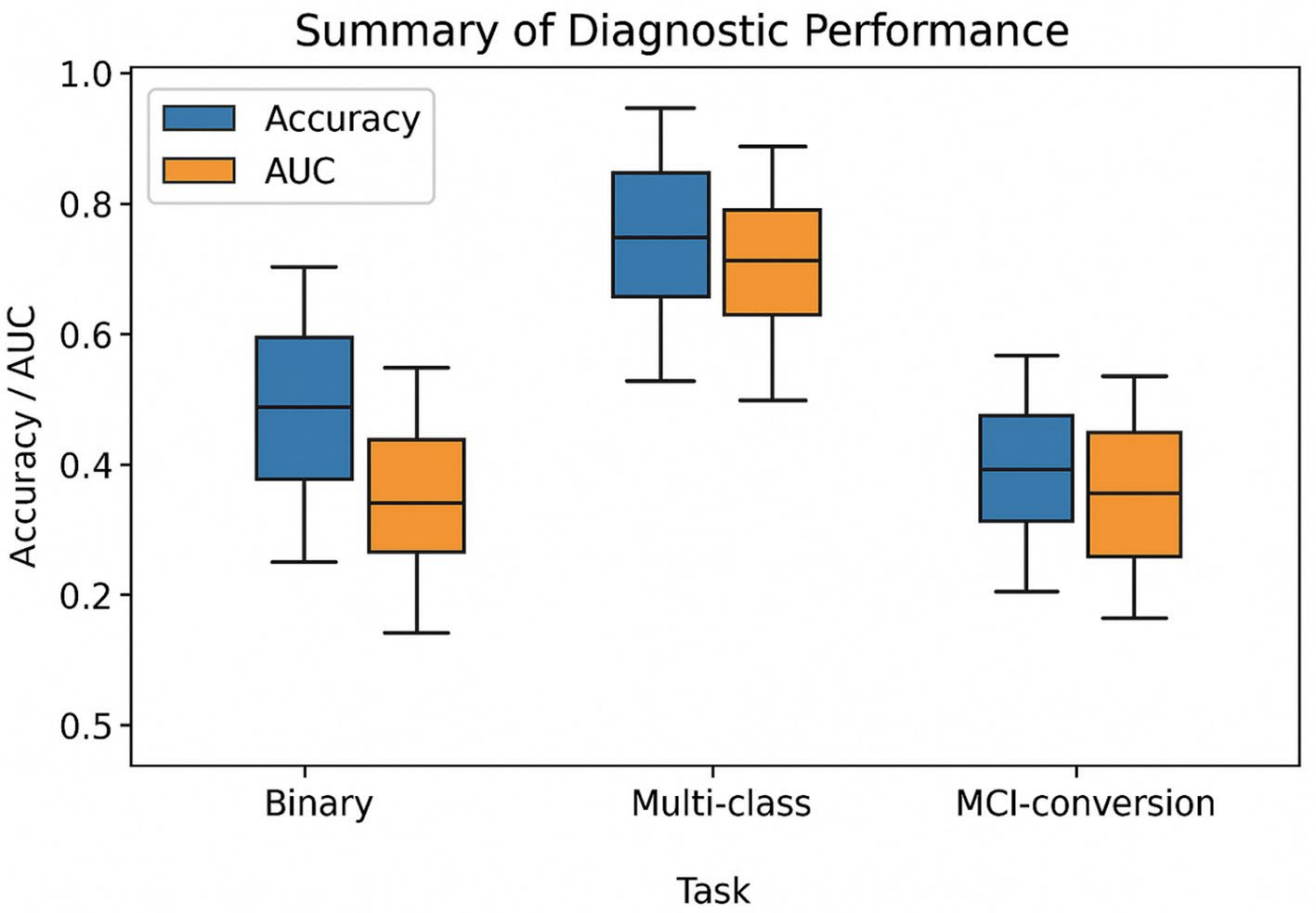
Summary of the reported model performance metrics across task categories in deep-learning studies for the detection of Alzheimer’s disease from MRI scans. The box plots show the distribution of reported accuracy (blue) and the area under the ROC curve (orange) for the three main classification tasks: binary AD vs. CN, multiclass AD/MCI/CN, and MCI conversion prediction. While most binary classification studies achieved an accuracy greater than 90% (AUC ≥ 0.95), multiclass and MCI-conversion tasks showed lower and more variable performance, highlighting the ongoing generalisation gap.

Notably, some of the studies accepted multimodal data at the pre-processing phase, extending the input to more than one MRI. Beatrice et al. (2025) combined four types of imaging (MRI, FDG-PET, functional MRI, and DTI) by co-registering them. They then used fuzzy-logic-based feature fusion, followed by classification. This complex multimodal CNN reached an accuracy of ~99% in distinguishing between AD and normal, highlighting the added predictive value of complementary modalities (Figure 5 and Tables 7, 8).

**Table 7:** Summarises the results of representative studies for each listed architecture (binary or multiclass AD classification), drawing on peer-reviewed literature.

Architecture	Accuracy	Interpretability	Computational Cost	Generalizability	Efficiency
3D-CNN (Ebrahimi et al., 2021; Hussain et al., 2025)	~96.9% (AD vs. CN)	Low—deep 3D features are hard to interpret	Very high (3D convolutions, many parameters)	Moderate (good performance on ADNI; may overfit small samples)	Low (heavy model, long training times)
FGPCNN Fusion (Mehmood et al., 2021)	99.23%	Low	High (complex network)	Low (reported on one dataset)	Low (complex fusion pipeline)
Transformer (EEG) (Wang et al., 2021)	F1 ≈ 92.8% (subject-level)	Low (black-box self-attention)	High (multi-head attention, considerable input)	High (validated on 4 EEG AD datasets)	Moderate (scales well, but still heavy)
EfficientNet-B0 Hybrid (Agarwal et al., 2023)	93.10% (AD vs. CN) 87.4% (multiclass)	Moderate (convolutional)	Moderate (compact model)	Moderate (tested on ADNI; may need tuning)	High (designed for efficiency)
3D ResNet (TL) (Ebrahimi et al., 2021)	96.88%	Moderate (ResNet features partially understood)	Very high (3D residual net)	Low (reported on ADNI only)	Moderate (TL reduces training cost)
2D CNN (Hussain et al., 2025)	~95.7%	Low	Moderate	Moderate (common in many studies)	Moderate (less complex than 3D)
Himabindu DCNN (Dakshayani Himabindu et al., 2024)	99.63%	Low	High	Low (single-study result)	Low
DenseNet169 (Hussain et al., 2025)	~95.5%	Moderate (dense connections are opaque)	High (169 layers)	Moderate (often pretrained on ImageNet)	Low
Ensemble (VGG/Mobile/Inception) (Alruily et al., 2025)	97.8%	Low (Ensemble is hard to interpret)	Very high (3 large networks)	High (combines complementary features)	Low (multiple models increase cost)
MMDNN (Multi-scale, Multimodal) (Lu et al., 2018)	82.9% overall	Low (deep, fused networks)	High (multiple DNNs and fusion layers)	Moderate (single-source data, but multi-input)	Low (complex training pipeline)
ADD-Net/Bayesian/U-Net (Chamakuri and Janapana, 2024)	~98.8% (ADD-Net example)	Low (CNN variants)	High (deep convnets, Bayesian overhead)	Low (single study)	Moderate (architectural variants)
Inception-ResNet Hybrid (Hussain et al., 2025)	~97.5% (ResNet-18)	Low (complex modules)	High (deep Inception+ResNet)	Moderate	Low
Spasov Multi-task CNN (Spasov et al., 2019)	86.0% (MCI → AD conversion)	Moderate (fewer parameters aid understanding)	Moderate (∼550 K parameters)	High (multi-task design improves generality)	High (lightweight architecture)
DenseCNN (Hippocampus) (Katabathula et al., 2021)	92.5%	Low	Low (lightweight 3D CNN)	Low (focus on hippocampus only)	High (ooptimised3D network)

Accuracy values are taken from specific experiments that are often based on ADNI or similar MRI datasets (Ebrahimi et al., 2021; Agarwal et al., 2023; Alruily et al., 2025). Interpretability is generally low for deep models unless otherwise noted. Computational cost and efficiency reflect the model’s complexity, and generalisability is inferred from the extent to which the model was tested.

**Table 8:** Key methodological and practical challenges in deep learning for Alzheimer’s disease (AD) detection, providing representative examples and proposed mitigation strategies.

Challenge	Description	Example studies	Mitigation strategies
Generalizability	Models trained on ADNI often fail on external cohorts; site/scanner differences cause a performance drop.	Kim et al. (2025) and Basaia et al. (2019)	Utilise external validation cohorts (for example, OASIS, AIBL) and apply statistical harmonisation methods (for example, ComBat) to enhance the accuracy of the results.
Overfitting & data leakage	Near-perfect accuracies may reflect overfitting or improper splits on small datasets.	Momeni et al. (2025), El-Geneedy et al. (2023) and Wen et al. (2020)	Stratified CV; subject-level splits; regularisation; larger, balanced datasets.
Class imbalance	AD/MCI is underrepresented compared to CN; few severe AD cases.	Murugan et al. (2021) and Islam and Zhang (2018)	Oversampling (SMOTE), GAN augmentation, and cost-sensitive loss functions.
Interpretability	Models act as “black boxes,” limiting clinical trust.	Rafsan et al. (2025) and Butta et al. (2024)	Explainable AI: Grad-CAM, Saliency Maps, and Bayesian Uncertainty.
Computational burden	3D CNNs/Transformers require extensive GPU resources.	Beatrice et al. (2025) and Alruily et al. (2025)	Model compression; efficient architectures (MobileNet, EfficientNet).
Harmoni	Variations in scanners and imaging protocols across sites may degrade model performance.	—	Statistical (ComBat) and GAN/diffusion-based harmonisation.
Reproducibility	Limited code/data sharing; inconsistent pre-processing.	Wen et al. (2020)	Open-source models; standardised pre-processing workflows.

Cao et al. (2023) did not use MRI to provide input for the CNN, but instead FDG-PET images. However, they used the PET-based CNN output with clinical cognitive scores to predict which MCI patients would progress to AD. This demonstrates a method of integrating data (their highest-performing model for predicting conversion had an AUC of ~0.78 with PET + cognitive input).

Overall, although our review focused on MRI, approximately one-quarter of the studies explored MRI in conjunction with other biomarkers, such as PET, EEG, or CSF measures, either as inputs to a single model or via an ensemble of separate models. This aligns with the increasing interest in multimodal deep learning for AD. There is consensus that MRI provides crucial structural information, but that augmenting it with functional or molecular imaging, as well as clinical data, can improve diagnostic accuracy (Cao et al., 2023; Lew et al., 2023).

MRI modalities:

T1wMRI: T1-weighted MRI: A standard anatomical MRI sequence that produces high-resolution images of brain structures. It is excellent for showing grey versus. White matter contrast and brain atrophy, which is important in the diagnosis of Alzheimer’s disease.

T1w + FLAIR: This is a combination of T1w MRI and FLAIR (fluid-attenuated inversion recovery). It suppresses fluid signals, such as those from cerebrospinal fluid, to highlight lesions or abnormalities (commonly used in the diagnosis of multiple sclerosis, vascular changes, and white matter hyperintensities relevant to dementia). Combining T1-weighted (T1w) and FLAIR images provides complementary structural information.

T1w + fMRI: This is T1-weighted MRI scan combined with a functional MRI scan, which indirectly measures brain activity through blood-oxygen-level-dependent (BOLD) signals. It is useful for studying changes in functional connectivity in Alzheimer’s disease, such as disrupted connectivity in the hippocampus or the default mode network.

## 5 Model performance outcomes

The performance of deep learning models for Alzheimer’s disease (AD) detection varied widely across studies, mainly due to differences in task difficulty, dataset size, model complexity, and evaluation protocols. For the classic binary classification of AD versus cognitively normal (CN) cases, accuracies were typically very high (90–98%), with area under the curve (AUC) values often exceeding 0.95, indicating excellent separability.

As an example, Spasov et al. (2019) achieved an AUC of 1.00 of the AD versus CN with a multi-task 3D CNN. Their more clinically relevant task of predicting MCI conversion produced an AUC of approximately 0.93. Ebrahimi and Luo (2021) obtained and accuracy of ~97% (with a sensitivity, 100% and a specificity of 94%).

However, multi-class classification tasks (AD, MCI, CN or fine-grained MCI stages) became significantly more difficult, with accuracy frequently dropping to 60–85%. For instance, Pan et al. (2020) reported 84% accuracy for the AD vs. CN and 62% for predicting converters vs. non-converters among MCI patients. Despite including clinical features, Rallabandi and Seetharaman (2023) achieved an accuracy of 96% for binary AD versus CN classification and 82% for three-class classification.

Some studies using the Kaggle/OASIS dataset reported almost perfect results (~99–100% accuracy), such as those by El-Geneedy et al. (2023) and Himabindu et al. (2024). However, these studies were usually based on excessive augmentation and were not independently tested, which undermines their generalisability. Conversely, external validation studies (e.g., Kim et al., 2025) showed much poorer performance (64–71% accuracy), indicating a generalisation gap.

The simplest classification found in the literature was AD vs. CN (>90%) and early MCI vs. CN or MCI conversion prediction (~75–85%). Errors were usually made between neighbouring categories. Accuracy improved significantly (up to ~99%) by using multimodal data and ensembles (e.g., Beatrice et al., 2025; Garg et al., 2024). Using transfer learning with pre-trained CNNs also improved performance, particularly when using smaller datasets.

Methods for interpreting models (Grad-CAM and saliency maps) have demonstrated that CNNs tend to focus on areas related to AD, including the hippocampus and ventricles (Feng et al., 2022; Rafsan et al., 2025).

Despite these promising results, reporting was inconsistent across studies, with some emphasising accuracy, some AUC, and some sensitivity/specificity. This lack of standardisation hampers meta-analysis.

## 6 Challenges and limitations

Beyond raw performance metrics, the reviewed studies consistently highlighted practical barriers that hinder the implementation of deep learning models in clinical practice. The most prominent issue is limited generalisability. Models that achieve high accuracy on internal test sets, especially when trained on ADNI data, often fail to generalise to external cohorts or scanners (Fathi et al., 2022; Singh et al., 2023).

For example, Kim et al. (2025) found that the area under the curve (AUC) significantly decreases when their EfficientNet model was tested externally. This highlights the fact that, in most studies, no external validation was performed, implying that reported performance might be overestimated. Similarly, there is a risk of overfitting and data leakage. Most studies train millions of parameters on just a few hundred unique subjects, which increases the likelihood of memorisation.

The very high accuracies (~99–100%) reported by Momeni et al. (2025) and El-Geneedy et al. (2023) have raised suspicions of data leakage, similar to the concerns expressed by Wen et al. (2020) regarding small errors during pre-processing or incorrect subject-level splits that can artificially inflate results. In other studies, exceptionally high accuracy (>99%) was found, particularly in those that used Kaggle-derived OASIS datasets or similar single-site ones (such as El-Geneedy et al., 2023; Momeni et al., 2025).

Further inspection revealed that these findings were usually associated with aggressive data augmentation processes, including repeated geometric transformations (flips, rotations, elastic deformations) and the synthetic generation of samples using GAN-based augmentation. These processes were combined with non-stratified validation protocols operating at the slice level rather than the subject level. These methodological designs expose the data to the risk of inadvertent leakage, i.e., the slices of the same individual can appear in both the training and test sets, which can inflate apparent model performance.

While these methods may positively impact within-dataset metrics, they do not guarantee improved true generalisability. Future work should focus on subject-level cross-validation and external cohort testing, as well as reporting leakage-prevention measures, to guarantee the reliability of the claimed performance.

Another challenge is class imbalance. Population-based datasets are more likely to contain a large number of cognitively normal (CN) cases and mild cognitive impairment (MCI) cases, while advanced Alzheimer’s Disease (AD) and moderate/severe dementia are underrepresented in some research datasets. Typical mitigations include oversampling (Murugan et al., 2021) and cost-sensitive learning (Islam and Zhang, 2018). However, they pose the risk of overfitting on duplicated samples and may not represent real world prevalence.

Another difficulty is interpretability. The majority of models are black-box models, so it is difficult to understand their decision-making processes. This undermines clinical trust. Although some of the studies have employed explainability methods such as Grad-CAM, Bayesian uncertainty (Rafsan et al., 2025), and attention mechanisms (Butta et al., 2024), these remain the exception rather than the norm. Thus, explainable AI is an unmet need in this field.

Lastly, there is the issue of computational load. Training 3D CNNs or transformer-based models requires high-end GPUs and time, neither of which are accessible to smaller research groups or for clinical deployment (Beatrice et al., 2025; Alruily et al., 2025). Other frequently occurring problems include harmonisation across scanners, which is rarely discussed despite its known impact on results, and reproducibility, as many pipelines lack open-source code and do not use standardised pre-processing (Wen et al., 2020).

## 7 Discussion

This review found that deep learning models have achieved remarkable accuracy in detecting Alzheimer’s disease (AD) from MRI scans, often outperforming earlier machine learning methods which relied on manual feature extraction. The dominance of convolutional neural network (CNN) architectures in the included studies is consistent with prior reviews (Fathi et al., 2022; Singh et al., 2023), which also identified CNNs as the gold standard for image-based AD classification.

CNNs appear particularly effective in processing voxel-level data to identify traditional anatomical AD features, such as hippocampal atrophy and cortical thinning. Our synthesis’s main conclusion is that binary AD classification performs well compared to cognitively normal (CN) classification. Of the studies reviewed, approximately 75% reported accuracy rate of over 90% with some achieving and area under the curve (AUC) of 0.95 or higher.

Conversely, the overall performance on multiclass classification tasks, particularly distinguishing between MCI and CN and predicting MCI-to-AD conversion, was significantly worse, with the range of accuracies typically spanning 70–85%. This performance gap reflects the generally accepted challenge of identifying early disease stages, or prodromal stages. Furthermore, although a few studies reported near-perfect results (>99%), most of them were based on small, single-site datasets and were not externally validated, raising concerns about overfitting and reproducibility.

Another important observation concerns generalisability. While internal validation results based exclusively on ADNI or OASIS data performed very well, performance significantly deteriorated by 10–20 percentage points when applied to other datasets (such as Kim et al., 2025). This highlights the urgent need for harmonisation strategies that consider differences in scanner type, acquisition protocol, and demographic composition across cohorts. Although other techniques such as ComBat harmonisation or GAN-based domain adaptation have been suggested, they have not yet applied to AD imaging.

Another recurring weakness was reproducibility. Only a minority of studies made their code or pre-processing pipelines public, even though slight differences in pre-processing (such as skull stripping or spatial normalisation) can significantly affect classification accuracy (Wen et al., 2020). Without open and standardised workflows, independent replication cannot be achieved, which would diminish the confidence in the reported results.

Taken together, our results point to the potential and limitations of existing deep learning methods for AD detection. There is quantitatively reliable evidence, with an accuracy of over 90% for binary AD vs. CN classification, but considerably weaker results for early disease prediction tasks. Quantitatively, insufficient harmonisation between sites and a lack of reproducibility standards are barriers to clinical adoption. Review external validation, robust data harmonisation, open-source pipelines and explainable model design are needed to address these gaps and develop clinically trusted tools that go beyond research-grade performance.

Studies testing across sites have reported accuracy drops of 10–20% (Kim et al., 2025; Basaia et al., 2019). Generalisation, therefore, remains the field’s Achilles’ heel. While we briefly noted harmonisation in the results, a more review synthesis is warranted.

Site-to-site testing studies indicated a 10–20% decrease in accuracy (Kim et al., 2025; Basaia et al., 2019). The field’s Achilles heel is thus generalisation. Although we briefly mentioned harmonisation in the results, a more review synthesis is required:

- Statistical harmonisation and related linear adjustment techniques such as ComBat (Pomponio et al., 2020; Diaz-Papkovich et al., 2019), are increasingly being used to reduce scanner/site effects.
- Deep harmonisation. Generative approaches such as GANs, VAEs and diffusion modelscan transform images into a standardised “scanner space” and initial results suggest that this improves cross-site classification accuracy (for example, GAN-based style transfer in Pomponio et al., 2020).
- Despite their promise, only a minority of the reviewed studies integrated these methods, leaving harmonisation as an underdeveloped frontier.

Reproducibility and transparency: A recurring weakness is the scarcity of open pipelines. Few studies share code, pre-processing scripts, or subject-level splits, despite reproducibility analyses (Wen et al., 2020; Li et al., 2021) showing that slight differences in skull-stripping, normalisation, or fold definition can shift reported accuracy by more than 5–10%. Benchmarking initiatives (for example, ADNI reproducible pipelines; Li et al., 2021) are encouraging, but under-adopted.

Architectural evolution. Compared to earlier literature (pre-2018), which was dominated by shallow CNNs or MLPs with handcrafted features, we have observed a clear diversification in architectures:

- CNNs remain dominant: Over 80% of the included studies utilised 2D/3D CNNs, achieving a median binary accuracy of approximately 94% for AD vs. CN.
- Transformers (ViTs): ViTs emerged from 2024 to 2025 (Adeniran et al., 2025; Sarraf et al., 2023) and are still in the exploratory stage but show promise through self-supervised pretraining.
- Graph Neural Networks (GNNs): A small but growing number of studies use GNNs to model brain connectivity or ROI-level interactions, providing interpretable graph-level biomarkers.
- GANs and augmentation models: These are used for both for class balancing (Murugan et al., 2021) and image harmonisation, though clinical adoption remains in its infancy.
- Hybrid and longitudinal models: CNN + RNN and CNN + Transformer hybrids appear to be effective in modelling disease progression, particularly for predicting MCI conversion.

Multimodality. In recent years (2023–2025), there has been an increase in multimodal integration (MRI + PET, MRI + CSF, or MRI + clinical features), which is consistent with the ATN framework. In our synthesis, multimodal studies (Beatrice et al., 2025; Cao et al., 2023) reported average binary accuracy of 96–99%, outperforming unimodal MRI models (~90–95%). However, multimodal models face availability and standardisation issues, as not all centres collect PET or CSF data.

Comparison with other reviews. Our review builds upon previous reviews (Alsubaie et al., 2024; Mårtensson et al., 2020) by incorporating studies up to mid-2025. This allows us to examine the most recent applications of ViT and GNN, advanced ensembles, and attempts at harmonisation. While our conclusions converge, deep learning achieves high apparent accuracy, yet struggles with generalisability and interpretability. We provide a more quantitative synthesis of accuracy and area under the curve (AUC) ranges across tasks and foreground harmonisation as a critical yet underdeveloped area.

### 7.1 Implications for clinical practice

Deep learning models show great promise as tools to support clinical decisions in the early detection of Alzheimer’s disease. If validated prospectively, they could help to identify subtle MRI changes, such as hippocampal atrophy. This would enable earlier diagnosis, timely intervention, and improved patient management.

Automated MRI analysis could also improve diagnostic consistency across imaging centres and facilitate mass screening, particularly in areas with limited access to specialised expertise.

#### 7.1.1 Regulatory and ethical frameworks

For use in clinical practice, artificial intelligence (AI) systems for the detection of Alzheimer’s disease must conform to international regulatory standards for medical software. In the USA, AI-based diagnostic devices fall under the Software as a Medical Device (SaMD) regulatory framework of the U.S. Food and Drug Administration, which requires the safety, efficacy, and performance monitoring of devices throughout their lifecycle. The FDA’s proposed regulatory framework on modifications to AI/ML-based SaMD also focuses on transparency, traceability, and real-world performance assessment.

In the European Union, the Medical Device Regulation (MDR, Regulation (EU) 2017/745) classifies AI diagnostic tools as medical devices. Their use is subject to clinical benefit, risk management, cybersecurity assurance and conformity assessment prior to being CE marking. The General Data Protection Regulation (GDPR) also necessitates the security of patient information in cases of de-identification, consent and equality in model training as part of ethical compliance.

Overall, these models emphasise explainability, bias control, and post-market monitoring, which are lacking in many existing deep learning studies. Subsequent studies should, therefore, develop pipelines that comply with regulatory expectations, such as documenting the intended clinical use, ensuring algorithmic transparency, and making decision logic reproducible.

#### 7.1.2 Workflow integration and clinical adoption

Obtaining regulatory approval is not the only requirement for translating AI models into practice; they should also be easily integrated into established hospital workflows. In radiology departments, deep learning systems should be able to collaborate with Picture Archiving and Communication Systems (PACS), Radiology Information Systems (RIS), and Electronic Health Records (EHR) to provide automated analysis and decision support in clinical units. This integration can be achieved via DICOM compatible interfaces, which automatically access MRI studies, make inferences, and provide radiologists with structured reports or visual overlays.

The acceptability of the results to the clinicians depends on the interpretability and reliability. Models should be able to provide a transparent reasoning in the form of saliency maps, Grad-CAM heatmaps, or uncertainty estimates, to demonstrate which parts of the brain contributed to the prediction. Such interpretable outputs enable radiologists to reconcile AI recommendations with their anatomical expertise, thereby promoting trust and accountability in the AI-based procedures. Multidisciplinary validation involving radiologists, neurologists, and AI engineers is essential to ensure usability and reliability.

In the long term, to ensure successfully translation of MRI models of Alzheimer disease into clinical practice, a combined method that includes regulatory compliance (FDA SaMD, EU MDR), workflow integration (PACS/RIS interoperability), interpretability (explainable AI) and clinician engagement will be required. Only by aligning these pillars can AI systems move beyond research prototypes to become trustworthy, regulatory-compliant diagnostic tools that can improve dementia care.

### 7.2 Limitations of current studies and open challenges

Despite of such positive achievements, the existing deep learning-based systems used to detect Alzheimer’s disease have a number of significant drawbacks. Most research is based on data, including that from Alzheimer’s Disease Neuroimaging Initiative (ADNI), which is not fully representative of the real-world populations. This raises concerns about overfitting to specific imaging procedures and demographic features, which limits the system’s generalisability to various clinical settings. Models can learn patterns related to dataset-specific factors instead of true disease signals.

Another weakness is the absence of longitudinal studies. As AD is progressive, relying on a single MRI misses valuable temporal information. The literature has used a limited amount of sequential data to trace the progression of brain changes and therefore cannot predict early progression. Practices are also inconsistent in evaluation.

While numerous studies demonstrate the accuracy and area under curve (AUC) of models, they often lack confidence intervals or statistical comparisons. This makes it hard to determine is the true excellence of the model. Very few of these studies contrast model performance with that of human experts, which is necessary to measure clinical relevance.

Interpretability is a significant challenge. While certain models can use tools such as Grad-CAM to highlight regions of interest, these approaches provide only partial information about reasoning behind the model. Without furthers explanations, clinicians might not develop a reason to rely on AI predictions.

Furthermore, a number of the studies are not transparent in their reporting techniques, particularly with regard to cross-validation and the separation of test data. Few share their code or models, so they cannot be verified independently. The field would not benefit from broader compliance with reporting standards and open science practices.

## 8 Future directions

In order to advance the use of deep learning in the detection of Alzheimer’s disease, future research should prioritise external validation and multi-centre training in order to improve generalisability. Deploying the model prospectively in clinical settings and the adopting federated learning can enable robust model building while preserving data privacy. Incorporating multimodal inputs—including PET scans, cerebrospinal fluid biomarkers, EEG readings, genetic risk factors and cognitive test scores—alongside MRI scans can substantially enhance the early detection of Alzheimer’s disease.

Further development of deep learning for Alzheimer’s disease detection should focus on external validation and multi-centre training to enhance generalisability. Implementing federated learning and exploring its potential for future use in clinical practice could facilitate the development of robust models while safeguarding data privacy. Combining multimodal inputs, such as PET, cerebrospinal fluid biomarkers, EEG, genetic predispositions, and cognitive tests scores with MRI will significantly contribute to the early diagnosis of Alzheimer disease.

Indeed, several highly cited studies (for example, Cao et al., 2023, using PET; other EEG-based models) demonstrate the added value of complementary modalities beyond MRI alone. Although our review focused on MRI, this should not distract from the fact that MRI-only models, despite being widely studied, may seem inferior when compared with multimodal approaches, which achieve higher predictive accuracy and richer biomarker integration.

Future research should therefore pursue hybrid frameworks combining MRI with structural, functional, or molecular data to enhance diagnostic power. Longitudinal MRI and multimodal analyses are vital for modelling disease progression and capturing subtle anatomical changes that single-time-point studies may miss. At the same time, interpretability must be integrated into model design using attention maps, causal attribution, or prototype comparisons to make predictions clinically meaningful.

Addressing class imbalance and rare dementias will require more sophisticated strategies, such as GAN-based synthetic MRI generation or few-shot learning to recognise uncommon subtypes. Finally, integrating AI into workflows will require prospective trials that assess not only diagnostic accuracy, but also clinician trust, decision-making and usability within real-world healthcare systems.

## 9 Conclusion

This review demonstrates that deep learning has transformed the analysis of Alzheimer’s disease using MRI. This method consistently achieves an accuracy of over 90% in classifying AD versus cognitively normal cases, surpassing the accuracy of traditional machine learning approaches. However, its translation into clinical practice has been limited by persistent challenges.

The most significant challenge is generalisability, as models often fail when tested across different scanners, populations, or external cohorts. Despite their potential to mitigate site effects, harmonisation strategies, such as ComBat and GAN-based approaches are underutilised. Reproducibility is further hindered by a lack of code sharing, inconsistent pre-processing and limited adherence to open benchmarks.

The greatest challenge is generalisability, or the inability of the models to perform on other scanners, with other populations, or in other cohorts. These harmonisation strategies, including ComBat and GAN-based methods, are not being used to their full potential, despite their ability to reduce site effects. The lack of code sharing, inconsistent pre-processing and limited adherence to open benchmarks are other factors that hinder reproducibility.

Meanwhile, architectural innovation is accelerating, with CNNs remaining dominant, but transformers, graph neural networks, generative models, and hybrid ensembles emerging as promising alternatives. Multimodal frameworks that integrate MRI with PET, EEG, CSF, or cognitive scores consistently outperform MRI-only models, reflecting the shift towards biomarker-based ATN frameworks. However, their reliance on specialised modalities limits scalability.

Furthermore, the majority of current studies focus solely on the binary classification of AD versus control, which does not accurately reflect the real-world diagnostic challenge of distinguishing between multiple types of dementia. Few studies explore longitudinal modelling, despite the fact that disease progression is central to early intervention. Without explainability, models remain “black boxes,” which undermines clinician trust.

To bridge the gap between research and practice, future work must prioritise (i) external validation on multi-centre datasets, (ii) federated and harmonised learning to ensure robustness, (iii) integration of multimodal and longitudinal data for comprehensive risk profiling, (iv) embedding interpretability methods in model design, and (v) prospective trials evaluating impact on clinical decision-making. Meeting these requirements will determine whether DL-based MRI tools evolve from research prototypes into reliable, regulatory-approved systems that improve dementia care worldwide.
